# Tuning the reflection band of ordered nanocomposites using direct laser writing

**DOI:** 10.1039/d5tc02985f

**Published:** 2026-06-18

**Authors:** Zengchun Xie, Jing Qian, Teodora Faraone, Conor Dillon, A. Louise Bradley, Larisa Florea, Colm Delaney

**Affiliations:** a School of Chemistry & AMBER, The SFI Research Centre for Advanced Materials and BioEngineering Research, Trinity College Dublin Dublin 2 Ireland CDELANE5@tcd.ie; b School of Physics and AMBER, The SFI Research Centre for Advanced Materials and BioEngineering Research, Trinity College Dublin Dublin 2 Ireland

## Abstract

Hierarchical ordering is common in Nature, where materials are organised in specific architectures across multiple scales, to achieve function-led design. Herein, we demonstrate multi-tiered organisation of silica nanoparticles (NPs), within a nanocomposite by employing direct laser writing (DLW). The hierarchical arrangement was optimised for wide-gamut reflected colour across the visible range by controlling NP size, NP type (core–shell and core-only), NP volume, and DLW fabrication parameters. The obtained microstructures were characterised using SEM, demonstrating retained order of non-close-packed NPs, and reproducible high-resolution fabrication with sub-micron features. We further used microspectroscopy to quantify the effect of fabrication parameters on structural colour, which is supported by FIB-SEM analysis, and exploited this effect to fabricate 3-dimensional multi-coloured images at the micro-scale. We demonstrate that this process can also be expanded to fabricate photonic nanocomposite microstructures using plasmonic core–shell NPs.

## Abstract 2

Direct laser writing of self-assembled silica nanoparticle composites enables the generation of microstructures with tuneable structural colour.

## Graphical abstract

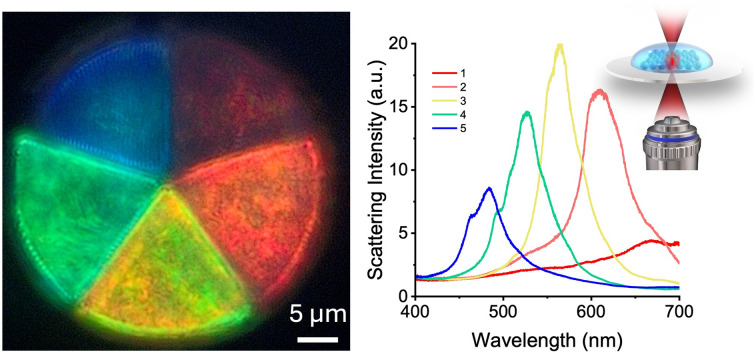

## Introduction

Self-assembly in Nature is observed in a multitude of instances, ranging from the structure of DNA to the formation of cellular membranes,^1,2^ and structural colouration.^3^ The latter arises from the constructive interference of light with ordered nanostructures.^4–6^ At its simplest, structural colouration is caused by a periodic variation in refractive index, and can be described by a combination of Bragg and Snell's laws.^7^ While many approaches have been explored for the realisation of artificial structural colour, nanoparticle self-assembly to achieve colloidal crystal assemblies (CCA) remains one of the most common approaches.^8,9^ Materials scientists have relied on scalable means of synthesising nanoparticles, such as the Stöber process (to generate silica nanoparticles) and emulsion polymerisation (to generate polymer nanoparticles).^10^ More recently, this approach has been extended to high-refractive-index materials such as titania, zinc sulphide, and copper oxide, broadening the optical functionality of CCAs.^11^

The advantages brought by embedding CCAs within a polymer matrix, in stabilising the colloidal structures, controlling their structural colour, and enabling new functions such as stimulus-response, cannot be overstated.^12^ Lee *et al.* incorporated silica nanoparticles into elastomeric polymers to demonstrate mechanochromic photonic devices. Under strain, the reflected colour of bulk films was modulated across the visible spectrum.^13^ Liao *et al.* used crosslinked polymeric nanoparticles within a soft poly(acrylamide-*co-N*-isopropylacrylamide) matrix for 3D printing of soft, responsive photonic structures.^14^ Embedding CCAs into polymerisable inks or photoresists suitable for 3D fabrication can further modulate inter-particle distance *via* hydration, stimulus-induced response of the host polymer matrix, or even fabrication parameters employed during the fabrication. This level of control over inter-particle distance after NP self-assembly was recently demonstrated through microfabrication of CCA-polymer composites using direct laser writing (DLW) *via* two-photon polymerisation.^15,16^

DLW using 2-photon polymerisation relies on multi-photon absorption of low-energy photons to initiate polymerisation.^17^ Outside the focal spot of the laser, the polymerisable material remains virtually transparent to the near infrared (NIR) irradiation, with polymerisation only occurring in a localised volume element, known as a voxel (∼300 nm × 100 nm). In 2021, Ritacco *et al.* demonstrated the possibility of using localised polymerisation to alter the ordering in a self-assembled photoresist. By changing the spacing between adjacent areas of high crosslinking density (voxels), they promoted the collapse of the ordered cholesteric liquid crystalline mesogens, resulting in modulation of photonic band gaps.^18^ In 2024, our group applied this approach to quasi-ordered nanocomposites for reinforcement and generation of photonic microstructures.^15^ The resulting reflection bands were broad and complicated by relative contributions from quasi-ordering and random scattering. More recently, we showcased that DLW can produce tuneable reflection bands by varying DLW parameters for monodisperse polymer nanoparticles within polymer networks.^16^

In this work, we highlight the universality of this approach by extending it to nanocomposites comprising monodispersed silica nanoparticles (both core-only and core–shell). Silica NPs of 144.7 ± 5.2 nm, 165.0 ± 4.9 nm, and 180.1 ± 9.1 nm were synthesised and incorporated in polyethylene glycol (PEG) based photoresists at 25, 30, and 35 vol%. Their microfabrication *via* DLW was investigated, with the largest reflection band range, obtained for the 35 vol% composition, showing a *λ*_max_ variation from 582 nm to 463 nm. This work is further expanded to Au core-silica shell nanoparticles, heralding the combination of plasmonic and photonic contributions.

## Experimental

### Materials

Phenylbis(2,4,6-trimethylbenzoyl) phosphine oxide (PBPO), poly(ethylene glycol) phenyl ether acrylate (PEGPEA), pentaerythritol triacrylate (Petri), Tween 80, tetraethyl orthosilicate (TEOS), ammonia solution 25% w/w, ethanol, hydrogen tetrachloroaurate(iii) trihydrate, sodium citrate dihydrate, 11-mercaptoundecanoic acid (MUA), were obtained from Sigma–Aldrich and used as received.

#### Silica NPs synthesis

Silica nanoparticles of varying diameters were prepared using a modified two-step seed-mediated growth method based on a previously reported procedure.^19^ Initially, silica seed particles were synthesised by mixing 20 mL of ethanol with 0.222 mL of TEOS (solution A), and separately preparing 20 mL of ethanol, 0.105 mL of deionised water, and 1.53 mL of 25% ammonia solution (solution B). Then solution A was added to solution B, and the reaction mixture was left at room temperature for 3 hours without stirring, yielding silica seed particles approximately 10–15 nm in diameter.

The growth of the silica NPs was performed *via* the Stöber process. For this, 14 mL of the seed dispersion was transferred into a mixture containing 116 mL of ethanol, 39 mL of deionised water, and 4 mL of 25% ammonia in a 250 mL conical flask under stirring. TEOS (200 µL per aliquot) was subsequently added to the mixture at 30 minute intervals to gradually grow the nanoparticles using a syringe pump. Dynamic light scattering (DLS) was used to monitor particle size during growth, and the TEOS addition was terminated once the desired hydrodynamic diameters (approximately 180 nm, 200 nm, and 220 nm) were achieved. After the addition of the final TEOS aliquot, the solution was stirred overnight to complete the reaction. The final silica nanoparticles were isolated by centrifugation in ethanol. The final concentration (in mg mL^−1^) was determined gravimetrically by drying and weighing a known volume (0.1 mL). Nanoparticle size and morphology were characterised by DLS and transmission electron microscopy (TEM).

#### 10 nm AuNPs

Gold nanoparticles with a diameter of approximately 10 nm were prepared following a modified citrate reduction method reported by Bastús *et al.*^20^ Briefly, 300 mL of 2.2 mM sodium citrate solution was added to a 500 mL three-neck round-bottom flask and heated to boiling under vigorous stirring using a heating mantle. A reflux condenser was attached to minimise solvent loss during the reaction. Once the solution reached a steady reflux, 1 mL of 50 mM HAuCl_4_ was rapidly injected. A visible colour transition from pale yellow to bluish-gray and finally to light pink occurred within 10 minutes, indicating the formation of gold nanoparticles. The synthesised AuNPs were characterised by TEM and UV-Vis spectroscopy to confirm particle size and optical properties.

#### Ligand exchange for 10 nm AuNPs

To improve the stability of gold NPs in ethanol and enhance interaction with silica, the surface ligands of citrate-stabilised 10 nm gold nanoparticles were replaced with 11-mercaptoundecanoic acid (MUA). For this, 10 mg of the as-prepared 10 nm AuNPs (in 300 mL of water) was heated to 60 °C under stirring. Then, 3.2 mg of MUA dissolved in 0.5 mL of ethanol was added dropwise. The reaction was allowed to proceed overnight to ensure complete ligand exchange. Excess MUA was removed by centrifugation. The resulting MUA-functionalised AuNPs were redispersed in 40 mL of Milli-Q water for further use.

#### Silica shell growth on 10 nm AuNP cores

To coat the MUA-functionalised AuNPs with a silica shell, 40 mL of the AuNP dispersion was mixed with 264 mL of ethanol, 8 mL of 25% aqueous ammonia, and 40 mL of deionised water in a 500 mL conical flask under stirring. Tetraethyl orthosilicate (TEOS, 0.1 mL per addition) was introduced *via* a syringe pump every 30 minutes. Dynamic light scattering (DLS) was used to monitor particle size. TEOS addition was stopped once the average hydrodynamic diameter reached approximately 220 nm. After the final TEOS aliquot, the solution was stirred overnight to complete the shell formation. The silica-coated gold NPs were purified by centrifugation in ethanol. The final NPs were stored in ethanol, and the concentration (in mg mL^−1^) was determined gravimetrically by drying and weighing a known dispersion volume (0.1 mL). Particle size and morphology were characterised by DLS, SEM, and TEM.

#### Dynamic light scattering (DLS)

DLS measurements were carried out using a Malvern Zetasizer Nano ZS with a 633 nm He–Ne laser. Samples were diluted in deionised water and measured in 1 cm path-length quartz cuvettes. The instrument operated in backscatter mode at 173°, and measurements were taken at 25 °C after a 60 s equilibration time.

#### Scanning electron microscopy (SEM)

SEM imaging was conducted using a Zeiss ULTRA Plus field emission scanning electron microscope. Prior to imaging, samples were coated with ∼15 nm of Au/Pd using a Cressington 208HR sputter coater (TED Pella Inc., 57 × 0.1 mm Au/Pd target) under argon for 20 s. ImageJ software was used to analyse particle size from SEM images, with at least 100 particles measured per sample.

#### Transmission electron microscopy (TEM)

TEM imaging was carried out using a JEOL JEM-2100 transmission electron microscope operated at an accelerating voltage ranging from 80 to 200 kV, depending on the resolution and contrast required. For sample preparation, 10 µL of the nanoparticle suspension was drop-cast onto a Formvar/carbon-coated copper grid (400 mesh, Ted Pella, 01754-F) and allowed to dry under ambient conditions. Grids were handled carefully to minimise contamination and ensure uniform particle distribution prior to imaging.

#### FIB imaging and analysis

Samples were affixed to stubs using conductive carbon tape. Prior to FIB milling, a thin layer of Au/Pd was sputtered onto the surface to minimise charging effects. Cross-sectional imaging was carried out using a Zeiss AURIGA dual-beam FIB-SEM system. The stage was tilted to 54° to align the sample at the eucentric height (5 mm working distance) for milling. FIB milling was conducted using a 30 kV Ga^+^ ion beam at a current of 240 pA. Subsequent SEM imaging of the exposed cross-sections was performed at 5 kV using a 30 µm aperture to achieve high-resolution contrast and minimise sample damage.

#### Photoresist preparation

The PC1, PC2 and PC3 photoresists were formulated by dispersing the respective NPs (S1, S2, S3) into a mixture composed of PEGPEA and Petri, with the NPs comprising 35 vol% of the total mixture. The resulting suspension was mixed and heated at 40 °C for 12 hours to slowly evaporate the ethanol. Following solvent removal, the photoinitiator PBPO was added (Table S1), and the mixture was sonicated thoroughly to ensure complete dissolution and uniform NP distribution. A similar protocol was used for the PC3-25 and PC3-30 photoresists, with the NPs comprising 25 vol% and 30 vol%, respectively.

At least 4 hours prior to fabrication, 10 µL of the unpolymerised photoresist was introduced into the printing cell. The cell consisted of a high precision top cover glass (12 mm diameter) and a silanised glass cover slip (22 mm diameter) on the bottom, separated by two strips of pressure-sensitive adhesive (PSA) of approximately 150 µm thickness. The cell was infiltrated with the photoresist by capillarity. This capillarity-driven filling facilitated the self-assembly of NPs within the photoresist, producing observable structural colour.

#### Direct laser writing fabrication

Three-dimensional nanocomposite microstructures were fabricated using two-photon polymerisation (2PP) with a commercial direct laser writing system (Photonic Professional GT2, Nanoscribe GmbH), using a femtosecond pulsed laser at 780 nm. The fabrication process was performed in oil-immersion mode with a 63× objective (Zeiss Plan-Apochromat, NA = 1.4, WD = 190 µm). Immersion oil (Zeiss Immersol) was applied directly onto the prepared cell.

Laser power (LP) was varied between 20–50 mW (nominal laser power: 40–100%, while scan speeds (SS) were used in the range of 3000–15 000 µm s^−1^. Following fabrication, the cover slip was carefully removed, and samples were developed in a 5% (v/v) Tween 80 solution in ethanol for at least 10 minutes at 60 °C to remove uncured resist. The resulting structures were inspected under optical microscopy to verify fabrication. To improve adhesion of the polymer structures to glass substrates, glass slides were sequentially cleaned with acetone, isopropanol, ethanol, methanol, and deionised water, dried under nitrogen, and treated with UV-ozone for 20 minutes. Slides were then functionalised with 3-(trimethoxysilyl)propyl methacrylate (3 vol% in ethanol, 0.1 vol% acetic acid) for 1 hour, rinsed with ethanol, and dried in an oven at 60 °C.

#### Microscopy and dark field measurements

Dark-field imaging was performed using an Olympus BX53 microscope equipped with a 20× objective lens (NA = 0.4, Olympus LMPlanFL N). Images were captured using a CCD camera, and scattering spectra were collected with an Andor Kymera 193 spectrometer using a ZL41 5.5 sCMOS Spectroscopy Camera. Scattering spectra were normalised using the formula: normalised spectrum = (*S*_sample_ − *S*_bg_)/(*S*_diffuser_ − *S*_bg_), where *S*_sample_ is the signal from the sample, *S*_bg_ is the ambient background signal, and *S*_diffuser_ is the reference signal measured from a solvent-filled glass cell on a ground glass diffuser.

#### Numerical simulations

The optical properties of the self-assembled system were studied using the Lumerical finite-difference time-domain (FDTD) solver. A 3 × 3 FCC (111) structure was built in the simulation domain, as shown in Fig. S14a. The FCC (111) region was meshed with a uniform grid size of 10 × 10 × 10 nm in all three directions. Six frequency-domain field and power (DFT) monitors (analysis group) were placed outside the meshed region to collect the electromagnetic response. A total-field scattered-field (TFSF) source was positioned between the DFT analysis group and the meshed structure to excite the system. Perfectly matched layer boundary conditions were applied in all three directions. The DFT monitor located in the reflection position was used to record the reflected signal.

## Results and discussion

**Fig. 1:**
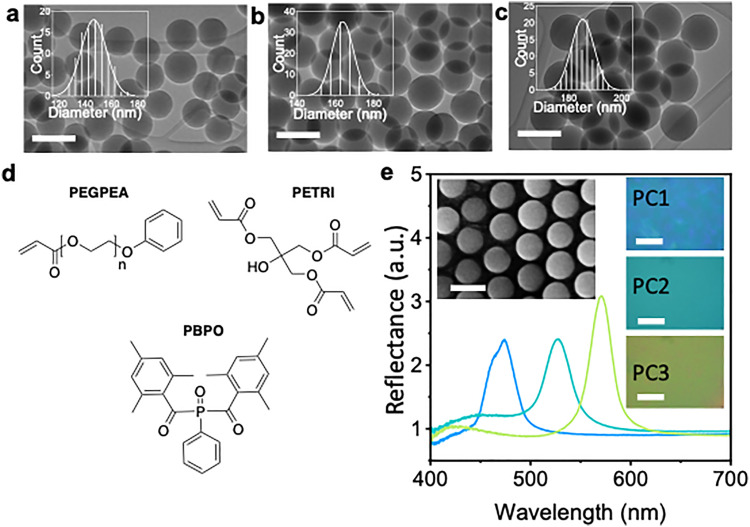
Nanoparticle and nanocomposite characterisation. TEM images of (a) S1, (b) S2, and (c) S3 NPs (Scale bar 200 nm). Inset shows the size distribution of particle diameter, as measured from TEM. (d) Components of photoresists. (e) Reflectance spectra of bulk PC1, PC2, PC3 samples. Insets show images of bulk samples (Scale bar is 1 mm) and the SEM image of PC2 bulk film (Scale bar 200 nm).

Three sizes of silica NPs were synthesised *via* a modified Stöber method.^19^ TEM revealed highly uniform and spherical particles, with average diameters of 144.7 ± 5.2 nm for S1, 165.0 ± 4.9 nm for S2, and 180.1 ± 9.1 nm for S3 (*N* = 100). DLS measurements showed *z*-average diameters of 181.5 ± 3.0 nm for S1, 208.0 ± 2.1 nm for S2, and 226.6 ± 6.4 nm for S3, with all samples exhibiting polydispersity index (PDI) values below 0.1, indicating excellent monodispersity. All NPs displayed negative zeta potential values below −20 mV, attributed to the presence of surface hydroxyl (–OH) groups, suggesting good colloidal stability. SEM further confirmed their high uniformity and size consistency with the TEM results (Fig. 1 and Fig. S1).

Using the synthesised silica NPs, namely S1, S2 and S3, three corresponding photonic composite (PC) photoresists, PC1, PC2, and PC3, were prepared at a concentration of 35 vol% NP. The other photoresist components were poly(ethylene glycol) phenyl ether acrylate (PEGPEA), the crosslinker pentaerythritol triacrylate (Petri), and photoinitiator phenylbis(2,4,6-trimethylbenzoyl) phosphine oxide (PBPO) (Fig. 1d and Table S1). Upon bulk polymerisation using white light, the resulting films exhibited distinct structural colours, as shown in Fig. 1e. These colours arise solely from variations in nanoparticle diameter. PC1, containing the smallest NPs of the series, displayed a reflection peak at *λ*_max_ = 474 nm. As the nanoparticle diameter increases, a corresponding redshift in the reflection band is observed: PC2 (containing S1) shows *λ*_max_ = 527 nm, while PC3, made with the largest NPs of the series, exhibits *λ*_max_ = 571 nm. SEM imaging of a PC2 film (inset of Fig. 1e) reveals a well-ordered nanoparticle arrangement, confirming successful NP self-assembly in the composite. Similar results were obtained for all PC bulk films.

DLW of all PCs showed successful microfabrication of 3D structures for a wide range of fabrication parameters. These included laser power (LP) and scan speed (SS), in addition to hatching (HD) and slicing distance (SL). Hatching and slicing distances refer to the adjacent spacing of polymerised voxels in *x*–*y* and *z* directions, respectively. In this work, HD of 0.2 to 1.4 µm and SL of 0.2 to 1.0 µm were employed for fabrication. Small distances between adjacent voxels result in higher voxel overlap, and therefore a stiffer polymeric structure, with minimised collapse. Conversely, increasing the distance of adjacent voxels translates to small voxel overlap, resulting in microstructures susceptible to collapse. Similarly, increasing the laser dosage (by increasing LP or decreasing SS) has been shown to result in polymer networks of higher crosslinking density and superior mechanical properties compared to when a lower dosage is used.^21,22^ Herein, LP was varied between 40% and 100% (where 100% corresponds to 50 mW), while SS was varied between 3 k and 15 k µm s^−1^.

**Fig. 2:**
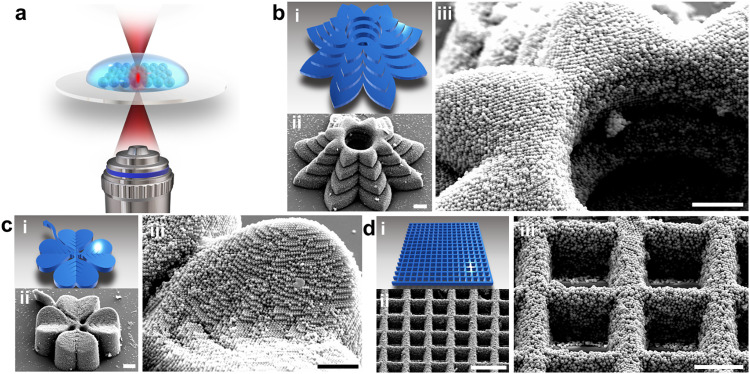
Schematic and results of direct laser writing (DLW) of 3D microstructures using silica NP-based photoresists. (a) Schematic illustration of the DLW setup incorporating silica NPs within the photoresist (PC3). (b) Fabrication of a lotus structure: (i) CAD model, (ii) SEM image of the printed lotus, and (iii) enlarged SEM image showing the long-range order of the silica NPs within the structure. The design dimensions were 60 × 60 × 20 µm, with fabrication parameters SL = HD = 0.2 µm, LP = 75%, and SS = 6000 µm s^−1^ (SE2, 10 kV). (c) Fabrication of a clover structure: (i) CAD model, (ii) SEM image of the printed clover, and (iii) enlarged SEM of a clover leaf showing ordered silica NPs. Design dimensions were 30 × 40 × 20 µm with SL = 0.5 µm, HD = 0.2 µm, LP = 50%, and SS = 10 000 µm s^−1^ (SE2, 10 kV). (d) Fabrication of a grid structure: (i) CAD model, (ii) SEM image of the fabricated grid, and (iii) enlarged SEM showing silica NPs. Design dimensions were 96 × 96 × 5 µm with SL = HD = 0.2 µm, LP = 60%, and SS = 10 000 µm s^−1^ (SE2, 5 kV). Scale bars: 6 µm for low-magnification SEM images (ii) and 3 µm for zoomed-in images (iii).

Fig. 2 shows a representative selection of 3D microstructures fabricated in PC3 *via* DLW (Fig. 2a), demonstrating the long-range order of self-assembled NPs within the composite. Fig. 2b–d show a series of structures which demonstrate a range of different design complexity, dimensionality, and feature sizes, comprising a representation of the 3D design, a low magnification scanning electron microscope (SEM) image highlighting the fidelity of the composite structure to the 3D design, and a higher magnification SEM micrograph, demonstrating the NP ordering on the nanoscale. Fig. 2b shows a layered flower structure (60 × 60 × 20 µm) where each petal layer has a thickness of 5 µm, giving a total flower height of 20 µm. Fig. 2c shows a four-leaf clover structure (30 × 40 × 20 µm), illustrating the ability to reproduce curved and intricate geometries with high fidelity, while highlighting excellent nanoscale ordering within the horizontal plane. Fig. 2d displays a grid structure (96 × 96 × 5 µm), demonstrating the fabrication of walls of ∼2 µm. These structures collectively demonstrate not only the versatility of the DLW process for complex 3D geometries but also the preservation of long-range silica NP order throughout the printed composite.

**Fig. 3:**
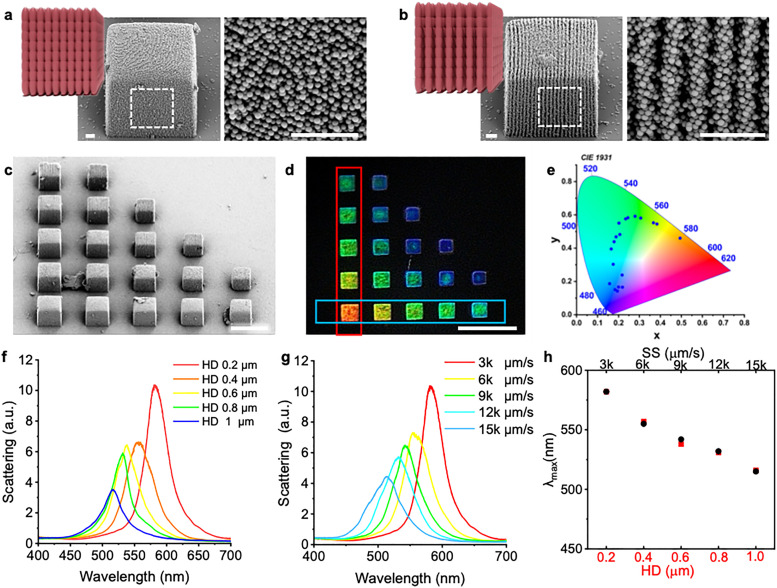
Design and fabrication of a microcube array using variation of SS and HD. (a) SEM images of a cube fabricated with small HD (0.2 µm) (scale bars: 2 µm) – inset shows a schematic of voxel overlap. (b) SEM images of cube fabricated with large hatching (0.8 µm) (scale bars: 2 µm); inset shows schematic of voxel spacing. (c) SEM image of the cube array fabricated at 40% laser power and SL 0.6 µm; *x*-axis represents SS from 3000 µm s^−1^ to 15 000 um s^−1^, while y-axis represents HD from 0.2 to 1 µm; (scale bar: 30 µm). (d) Corresponding dark-field microscopy image of a 5 by 5 cube array hydrated in deionised water (scale bar: 50 µm). (e) CIE 1931 chromaticity diagram corresponding to the microcube array in (d). (f) Scattering spectra of selected cubes highlighted by the red box in (d). (g) Scattering spectra of selected cubes highlighted by the blue box in (d). (h) *λ*_max_ (nm) *versus* scan speed or hatching distance.

Although all three photonic composites (PC1, PC2 and PC3) were subjected to DLW using the same optimised parameters, at 35 vol% NP loading, only PC3 exhibited a broad and vivid colour range in the resulting 3D microstructures (Fig. 3 and Fig. S2). This observation highlights the critical role of nanoparticle size in tuning the photonic bandgap. As shown in Fig. S2, the smaller nanoparticles (used in PC1 and PC2) limit the achievable periodicity necessary to shift the photonic bandgap across the visible spectrum using DLW parameter changes. Consequently, structures fabricated from PC1 and PC2 display narrower range and blue-shifted colour compared to PC3. In contrast, the larger nanoparticles (S3, diameter_TEM_ = 180.1 ± 9.1 nm) in PC3 allowed for a wider range of tuneable bandgaps, spanning a broader portion of the visible spectrum (Fig. 3).

We further investigated the effect of nanoparticle volume fraction on the structural colour of the microstructures by incorporating S3 NP in the same photoresist formulation, but at 25% and 30 vol% NP. The same 5 × 5 microcube array was fabricated in all three S3-containing photoresists, at 25%, 30% and 35 vol%, respectively, using a variation in HD from 0.2 to 1 µm (in 0.2 µm increments), and SS from 3000 to 15 000 µm s^−1^ (in 3000 µm s^−1^ increments), while maintaining SL = 0.6 µm and LP = 40%. As shown in Fig. S3 reducing the nanoparticle content from 35 vol% (PC3) to 30% (PC3-30) and 25 vol% (PC3-25) resulted in a noticeable red shift in the reflected colour. However, PC3-25, the photoresist with the smallest S3 NP vol% of the series, showed a reduced fabrication parameter range, compared to PC3 and PC3-30 (Fig. S3 and Fig. 3c and d). As a result, only 11 of the micro-cubes in the array were successfully fabricated for PC3-25, compared to 19 for PC3-30 and PC3. Successful fabrication in PC3-25 required higher laser dosage (through reduced SS values), and/or high voxel overlap (through reduced HD values). This also supports our previous result, where silica NPs also contribute to mechanical reinforcement, with higher silica NP content expanding the DLW parameters for successful microfabrication.^15^ When comparing the spectra for microcubes fabricated at SS = 3000 µm s^−1^ and HD = 0.4 µm, under the same conditions, we observe *λ*_max_ values of 557, 635, and 648 nm for the 35%, 30%, and 25 vol% samples, respectively, confirming the overall redshift trend with decreasing nanoparticle volume fraction.

Given the superior performance obtained for PC3 in terms of colour gamut and fabrication range, this photoresist was selected for all subsequent experiments in this study. This value is above the self-assembly threshold for the S3 nanoparticles, which is estimated to be approximately 25 vol%, ensuring robust structural colouration and mechanical fidelity of the composite microstructures.

Following the optimisation of nanoparticle volume fraction, we investigated the effect of microstructure height on the resulting colour. As shown in Fig. S4, the DF scattering spectra (Fig. S4b) demonstrate that increasing the height of the microstructure from 5 to 60 µm significantly enhances the intensity of the reflection band, while the peak position (*λ*_max_) remains effectively unchanged. To systematically investigate how structural colour in the fabricated microstructures is influenced by key fabrication parameters, we further studied the effects of HD, SL, SS, and LP. For this purpose, two parameters were fixed while the remaining two were varied to isolate their respective contributions. The most extensive colour gamut was achieved by fixing SL at 0.6 µm and LP at 40%, as shown in the dark-field image in Fig. 3d. The corresponding SEM image of the printed microcube array (Fig. 3c) confirms high structural fidelity and precision in the 3D fabrication process. The hatching distance, which defines the centre-to-centre spacing of adjacent voxels in the *xy*-plane, plays a critical role in determining the nanoparticle density and spacing, as illustrated in Fig. 3b.

The structural colour of micro-cubes, fabricated through variation of HD (0.2 µm to 1 µm) and SS (3 k to 15 k µm s^−1^) at constant LP (40%) and SL (0.6 µm), spans a broad region of the CIE 1931 chromaticity diagram, as shown in Fig. 3e, reflecting the tuneability of optical properties *via* fabrication parameters. Spectral analysis reveals that increasing the hatching distance, as shown in Fig. 3f, or increasing the scan speed, as shown in Fig. 3g, both result in a blue shift of the bandgap. In addition to the microcube array, we also designed and fabricated a heart-shaped array using the same fabrication parameters to further validate the observed trends (Fig. S5). The printed heart structures also exhibit a broad colour range and reveal the same *λ*_max_ trend when changing hatching distance and scan speed.

The effect of slicing distance and hatching distance on structural colour was further investigated using a combinatorial parameter array (Fig. S6). These parameters govern the spatial overlap between individual laser voxels in the *z* and *xy*-directions, respectively, and thus affect the effective nanoparticle volume fraction (vol%) in the final structure. As shown in Fig. S6b, the dark-field microscopy image of the micro-cube array revealed strong variations in colour across the studied parameter space. The smallest HD and SL values (0.2 µm) produced colours in the red region. This trend was confirmed by the scattering spectra (Fig. S6c and f) and *λ*_max_ plots (Fig. S6d and g), where a red shift was recorded for microcubes fabricated at lower HD and SL. This behaviour can be attributed to increased voxel overlap, which leads to a higher degree of polymerisation per unit volume, minimised collapse of the ordered nanocomposite, and results in a band gap comparable to that found in the bulk composite material.

Similarly, we investigated the influence of laser power and scan speed on the structural colour response of the fabricated nanocomposite arrays (Fig. S7). The bright-field image (dried state) and dark-field image (hydrated state) both show vivid structural colours. Since the structures do not swell measurably in water, the colours remain relatively unchanged after hydration. As scan speed increases (with LP fixed at 40%), a distinct blue shift in the reflectance peak is observed (Fig. S7c). This trend is further supported by the corresponding CIE plot (Fig. S7e). Interestingly, when LP is varied from 40% to 100% at a fixed scan speed of 3000 µm s^−1^, only a minimal *λ*_max_ shift was observed (Fig. S7f–g). This suggests that at low scan speeds, further increase of LP does not significantly affect the local refractive index or nanoparticle spacing. The laser power (LP) vs hatching distance (HD) array in Fig. S8 shows a clear red shift with increasing LP at fixed HD (Fig. S8f–g). This occurs because higher LP at this HD increases the energy density per unit area. Tuneable colour printing has been reproduced across multiple photoresists, fabrication steps and substrates, with good reproducibility, as highlighted in Fig. S9–S11, Table S2. For example, micro-cubes fabricated at constant parameters of slicing distance (SL) = 0.4 µm, hatching distance (HD) = 0.2 µm and laser power (LP) = 40%, while varying the scan speed (SS), from 3000 µm s^−1^ to 15 000 µm s^−1^ in 3000 µm s^−1^ increments, returned *λ*_max_ values of 587.1 ± 5.6 nm (*n* = 4) for SS = 3000 µm s^−1^; 564.1 ± 2.1 nm (*n* = 4) for SS = 6000 µm s^−1^; 553.8 ± 4.0 nm (*n* = 4) for SS = 9000 µm s^−1^; 544.8 ± 3.8 nm (*n* = 4) for SS = 12 000 µm s^−1^; and 538.9 ± 1.8 nm (*n* = 4) for SS = 15 000 µm s^−1^ (Fig. S9, Table S2). Colour variation across single micro-structures also shows relatively good homogeneity for optimised fabrication parameters, as evidenced by the microscopy images. For example, a cube fabricated with LP = 40%, HD = 0.2 µm, SL = 0.6 µm, and SS = 3000 µm s^−1^, was used for comparing spectra taken in different areas of the cube (*i.e.* total area, left half, right half), and returned a *λ*_max_ average value of 586.5 ± 5.1 nm (*n* = 3), with good spectral overlap, as shown in Fig. S10. As typically the entire area of the cube (20 × 20 µm) was used for spectrum recording, inhomogeneous colours resulted in broad spectral profiles, exhibiting discernible shoulder features. This is particularly the case for fabrication parameters that resulted in inhomogeneous structural collapse, such as low LP, high SL, HD, or SS, close to the critical exposure dose.

To further investigate the internal structural changes in the nanocomposite induced by varying the hatching distance (HD), a parameter of choice for inducing colour changes in this work, we performed focused ion beam scanning electron microscopy (FIB-SEM) on selected regions of the microcube array shown in Fig. S12a. Cross-sectional FIB-SEM images from structures fabricated at a constant LP of 60% but with increasing HD (from 0.2 µm to 1 µm) revealed notable differences in internal morphology. Increasing HD appears to result in a more closely-packed array, caused by the collapse of the structure. While FIB-SEM images of individual slices can offer insight into NP assembly, demonstrating long-range ordering, FIB-SEM tomography of consecutive slices (>6/particle) and particle tracking algorithms are required to truly understand the position and orientation of each NP in the assembly and quantitatively estimate the differences in interparticle distance between the samples fabricated at different HD, or through variation of other fabrication parameters.^23^ Nevertheless, the optical spectra of some of the PC3 microstructures produced in this work (*i.e.*Fig. 3f, HD > 0.4 um, Fig. 3g, SS > 6000 µm s^−1^; and Fig. S5, S6 and S13), do show *λ*_max_ values smaller than what was obtained for the bulk photopolymerised film (Fig. 1e, *λ*_max_ (bulk PC3 film) = 571 nm). For example, *λ*_max_ values of 520 nm and 525 nm (Fig. 3f), were obtained for microstructures fabricated at HD = 1 µm and 0.8 µm, respectively (SS = 3000 µm s^−1^; LP = 40%; SL = 0.6 µm). This indicates that inter-particle distance is further reduced through DLW fabrication, either post-development, through structural collapse, as supported by prior work,^15,16^ or through the generation of a depletion zone during DLW, driving the particles closer together.^24^ FDTD simulations using a FCC(111) lattice model, in which the (111) planes are stacked along the *z*-direction demonstrate that both nanoparticle diameter and nanoparticle concentration in the nanocomposite photoresist play critical roles in determining the resulting structural colours (Fig. S14). Specifically, larger nanoparticle diameters and lower nanoparticle concentrations increase the interlayer spacing between (111) planes, leading to a redshift in the reflection spectra, as previously demonstrated by us.^16^ The influence of refractive index (Fig. S14e and f) was also simulated, demonstrating that increasing the refractive index of either the nanoparticles or the surrounding polymer matrix results in a gradual redshift of the spectra. This effect is more pronounced when varying the polymer refractive index. Additionally, a higher refractive-index contrast enhances the spectral intensity. However, for small refractive-index changes (*e.g.*, Δ*n* ≈ 0.05), the corresponding spectral shift is negligible.

**Fig. 4:**
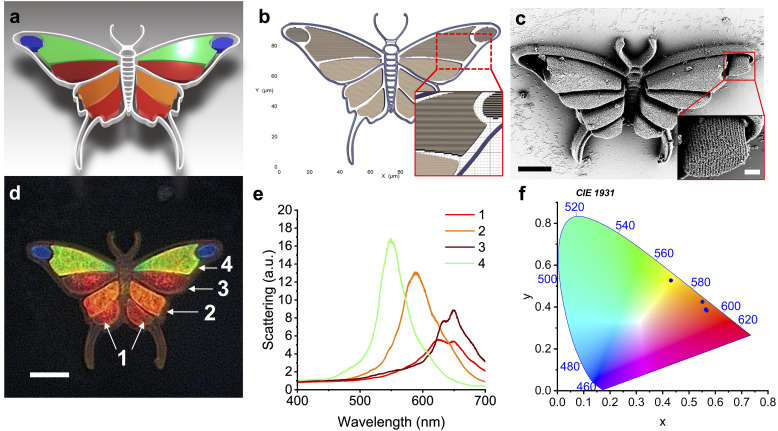
Design and fabrication of a butterfly microstructure displaying different colours by changing SL and HD employed during fabrication. (a) 3D model. (b) STL file of the butterfly shows the variation of slicing and hatching. (c) SEM images of the butterfly structure (scale bar: 20 µm) with magnification inset (scale bar: 3 µm) showing upper wing detail. (d) Dark-field microscopy image of the butterfly hydrated in deionised water (scale bar: 20 µm). (e) Corresponding scattering spectra of the 4 parts of the butterfly printed at LP 60%, SS 10 000 µm s^−1^. (f) The CIE 1931 chromaticity diagram corresponding to the 4 parts. Fabrication parameters: region 1: SL = HD = 0.2 µm, region 2: SL = 0.4 µm, HD = 0.2 µm, region 3: SL = 0.2 µm, HD = 0.4 µm, region 4: SL = 0.6 µm, HD = 0.6 µm.

Having determined the required DLW parameters for colour variation, we demonstrate the fabrication of a multi-coloured microstructure using a single nanocomposite photoresist (PC3 at 35 vol%), as shown in Fig. 4. The design began with a 2D digital design, which was segmented into five regions, each representing a different target colour. These segments were individually converted into STL files and imported into the DLW software (DeScribe), where specific slicing distance and hatching distance parameters were assigned based on the earlier parameter study. Programming inter-line and inter-layer voxel distances enabled precise control over the structural colour of each region. During fabrication, each segment was fabricated in a programmed sequence, allowing not only SL and HD variation but also LP and SS. As shown in the dark-field image (Fig. 4d), this method successfully generated distinct colours by parameter tuning rather than changes in material composition. The corresponding scattering spectra (Fig. 4e) reveal distinct scattering peaks for the different regions (the spectrum from the blue region could not be recorded due to its small size). The multi-coloured design shown in Fig. 4a–d was reproduced across multiple fabrication steps to produce eight butterfly structures shown in Fig. S11, demonstrating the reproducibility of the colour printing approach described herein. Similarly, we designed and fabricated a five-part colour wheel to further demonstrate the spatial control over structural colour enabled by tuning the fabrication parameter (Fig. S13). Each part of the colour wheel was designed using a distinct combination of slicing and hatching distances to yield a different colour (Table S3). The dark-field microscopy image reveals vivid and well-defined colours across the five regions, and the corresponding scattering spectra showed distinct peaks for each region.

To further explore the integration of plasmonic functionality into self-assembled photonic structures, we synthesised Au@SiO_2_ core–shell nanoparticles using a modified Stöber method. TEM confirmed the successful encapsulation of gold cores within uniform silica shells. SEM analysis showed a narrow size distribution with an average diameter of 167 ± 6.8 nm (*N* = 100), while DLS revealed a hydrodynamic diameter of ∼206 nm. Zeta potential measurements confirmed strong colloidal stability in aqueous dispersion, attributed to the negatively charged silica surfaces (Fig. S15).

Although these Au@SiO_2_ nanoparticles are similar in size to the S2 silica nanoparticles, the Au@SiO_2_-based structures exhibit different optical behaviour. When we fabricated the same structural designs used for PC3, the Au@SiO_2_ nanocomposite produced significantly broader and more intense colour (Fig. S16). This difference suggests that the presence of the plasmonic gold core may play a critical role in enhancing the colour tuneability in DLW-fabricated structures. These findings highlight the potential of controlling coupling between particles to modulate their collective behaviour. When further combined with responsive materials this work holds promise for applications in plasmon-enhanced biosensing, high-resolution colour printing, responsive optical devices, and nonlinear optics.

## Conclusion

In this study, we present a flexible method for fabricating wide-gamut structural colours using silica-based photoresists combined with direct laser writing. Through the precise synthesis of monodisperse silica nanoparticles and Au@SiO_2_ core–shell nanoparticles, we determined the nanoparticle diameter and volume thresholds that are essential for achieving ordered self-assembly and structural colouration. Using SEM, microspectroscopy, and FIB-SEM techniques, we systematically studied the effects of DLW fabrication parameters (including hatching distance, slicing distance, scan speed, and laser power) on structural colour and photonic bandgap. Our results indicate that larger nanoparticle sizes (S3 diameter_TEM_ = 180.1 ± 9.1 nm), such as those employed in PC3, enable a broader range of colour tuneability compared to S1 and S2 (S1 diameter_TEM_ = 144.7 ± 5.2 nm; S2 diameter_TEM_ 165.0 ± 4.9 nm), thus allowing colour control across the full visible spectrum.

Programmable structural colour was established using a single material composition, achieved by adjusting fabrication parameters. This approach allowed us to produce multi-colour 3D microstructures without changing the photoresist composition. This high level of spectral control demonstrates the strong potential of DLW in additive manufacturing of photonic materials with high resolution. By integrating the adaptability of the Stöber process with programmable DLW, this work provides a pathway toward next-generation nanocomposites with hybrid photonic–plasmonic functions and highly tuneable colours. Scalability of this approach remains a challenge that could be at least partially addressed by progress in DLW such as two-photon grayscale lithography. Additionally, thorough 3D characterisation of microstructure internal NP ordering is needed in order to truly understand the effect of DLW parameter variation on ordering and interparticle distance in *x*, *y*, and *z* directions. While the spectral trends are well documented, they underscore an important next step toward fully understanding how DLW parameters influence internal nanoparticle ordering in all spatial directions.

## Conflicts of interest

The authors declare no conflicts of interest.

## Supplementary Material

TC-014-D5TC02985F-s001

## Data Availability

The authors declare that the main data supporting the findings of this study are available within the article and its supplementary information (SI). Supplementary information: DLS and SEM data for synthesised NPs, compositions of photoresists for DLW, spectral data and analysis to support the main manuscript, and FDTD simulations. See DOI: https://doi.org/10.1039/d5tc02985f. Raw spectral data as well as SEM, TEM and optical microscopy images are available from Zenodo at DOI: https://doi.org/10.5281/zenodo.20387105 or from the corresponding authors upon reasonable request.

## References

[cit1] Seeman N. C. (1982). J. Theor. Biol..

[cit2] Pohorille A., Deamer D. (2009). Res. Microbiol..

[cit3] Burg S. L., Parnell A. J. (2018). J. Phys.: Condens. Matter.

[cit4] Lloyd V. J., Nadeau N. J. (2021). Curr. Opin. Genet. Dev..

[cit5] Jeon D.-J., Paik S., Ji S., Yeo J.-S. (2021). Appl. Microsc..

[cit6] Teyssier J., Saenko S. V., Van Der Marel D., Milinkovitch M. C. (2015). Nat. Commun..

[cit7] Fan N., Zhou C., Myagkaya E. (2021). Sci. Rep..

[cit8] Cai Z., Li Z., Ravaine S., He M., Song Y., Yin Y., Zheng H., Teng J., Zhang A. (2021). Chem. Soc. Rev..

[cit9] Li Z., Fan Q., Yin Y. (2022). Chem. Rev..

[cit10] Stöber W., Fink A., Bohn E. (1968). J. Colloid Interface Sci..

[cit11] Geng J., Shao K., Zhang P., Chen C., Huang S. (2025). J. Biotechnol..

[cit12] Zhu K., Fang C., Pu M., Song J., Wang D., Zhou X. (2023). J. Mater. Sci. Technol..

[cit13] Lee G. H., Han S. H., Kim J. B., Kim J. H., Lee J. M., Kim S.-H. (2019). Chem. Mater..

[cit14] Liao J., Ye C., Guo J., Garciamendez-Mijares C. E., Agrawal P., Kuang X., Japo J. O., Wang Z., Mu X., Li W., Ching T., Mille L. S., Zhu C., Zhang X., Gu Z., Zhang Y. S. (2022). Mater. Today.

[cit15] Augustine A., Qian J., Faraone T., Kolagatla S., Prochukhan N., Morris M. A., Bradley A. L., Florea L., Delaney C. (2024). Small.

[cit16] Faraone T., Qian J., Kolagatla S., Bradley A. L., Florea L., Delaney C. (2025). Adv. Mater..

[cit17] O’Halloran S., Pandit A., Heise A., Kellett A. (2023). Adv. Sci..

[cit18] Ritacco T., Aceti D. M., De Domenico G., Giocondo M., Mazzulla A., Cipparrone G., Pagliusi P. (2022). Adv. Opt. Mater..

[cit19] Reinhardt N., Adumeau L., Lambert O., Ravaine S., Mornet S. (2015). J. Phys. Chem. B.

[cit20] Bastús N. G., Comenge J., Puntes V. (2011). Langmuir.

[cit21] Sedghamiz E., Liu M., Wenzel W. (2022). Nat. Commun..

[cit22] Song D., Husari A., Kotz-Helmer F., Tomakidi P., Rapp B. E., Rühe J. (2024). Small.

[cit23] Van Der Hoeven J. E. S., Van Der Wee E. B., De Winter D. A. M., Hermes M., Liu Y., Fokkema J., Bransen M., Van Huis M. A., Gerritsen H. C., De Jongh P. E., Van Blaaderen A. (2019). Nanoscale.

[cit24] Matter F., Luna A. L., Niederberger M. (2020). Nano Today.

