# Supplementary material for: Tuning the reflection band of ordered nanocomposites using direct laser writing
Source: J Mater Chem C Mater. 2026 Jun 18. Online ahead of print. doi: 10.1039/d5tc02985f (PMC13322751; doi:10.1039/d5tc02985f)
Supplement: TC-OLF-D5TC02985F-s001 [file TC-OLF-D5TC02985F-s001.pdf]

Supplementary Information (SI) for Journal of Materials Chemistry C.
This journal is © The Royal Society of Chemistry 2026

Tuning the Reflection Band of Ordered Nanocomposites using Direct Laser Writing

Zengchun Xie,[1] Jing Qian,[1] Teodora Faraone,[1] Conor Dillon,[1] A. Louise Bradley,[2] Larisa Florea,[1] Colm Delaney[1]*

[1]School of Chemistry & AMBER

The SFI Research Centre for Advanced Materials and BioEngineering Research

Trinity College Dublin

Dublin 2, Ireland

E-mail: CDELANE5@tcd.ie

[2]School of Physics and AMBER

The SFI Research Centre for Advanced Materials and BioEngineering Research

Trinity College Dublin

Dublin 2, Ireland

a

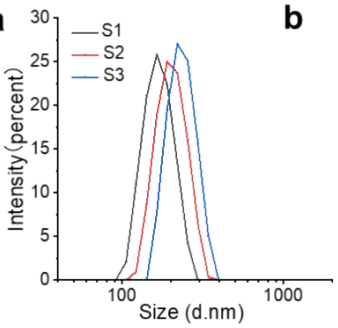

b

Sample	S1	S2	S3
DLS/nm	181.5±3.0	208.0±2.1	226.6±6.4
PDI	0.049	0.068	0.03
Zeta potential (mV) in water	-24.3±2	-31.7±1.4	-22.9±2.1
Size distribution by TEM/nm	144.7±5.2	165±4.9	180.1±9.1
Size distribution by SEM/nm	146.5±9.7	163.9±5.6	185.3±4.3

c d e

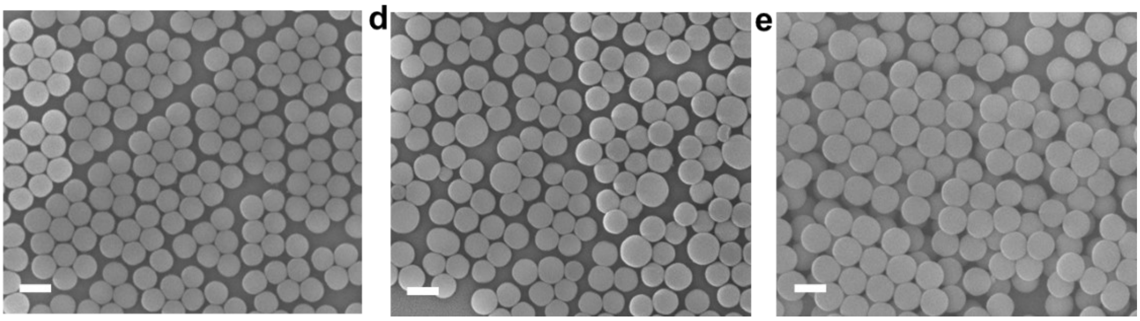

Figure S1. Characterisation of S1, S2, and S3 NPs. a) Overlapped DLS curves by intensity. b) Physicochemical properties summary table. c–e) SEM images of c) S1, d) S2, and e) S3 NPs. Scale bar 200 nm.

Table S1. Photoresist composition.

Compound	Molecular weight (g/mol)	Amount (mg)	Volume(μL)		
			25 vol%	30 vol%	35 vol%
NPs		100	50	50	50
PEGPEA	324	82	118	91.5	72
Petri	298.29	23.5	32	25	20
PBPO	418.5	3.4			

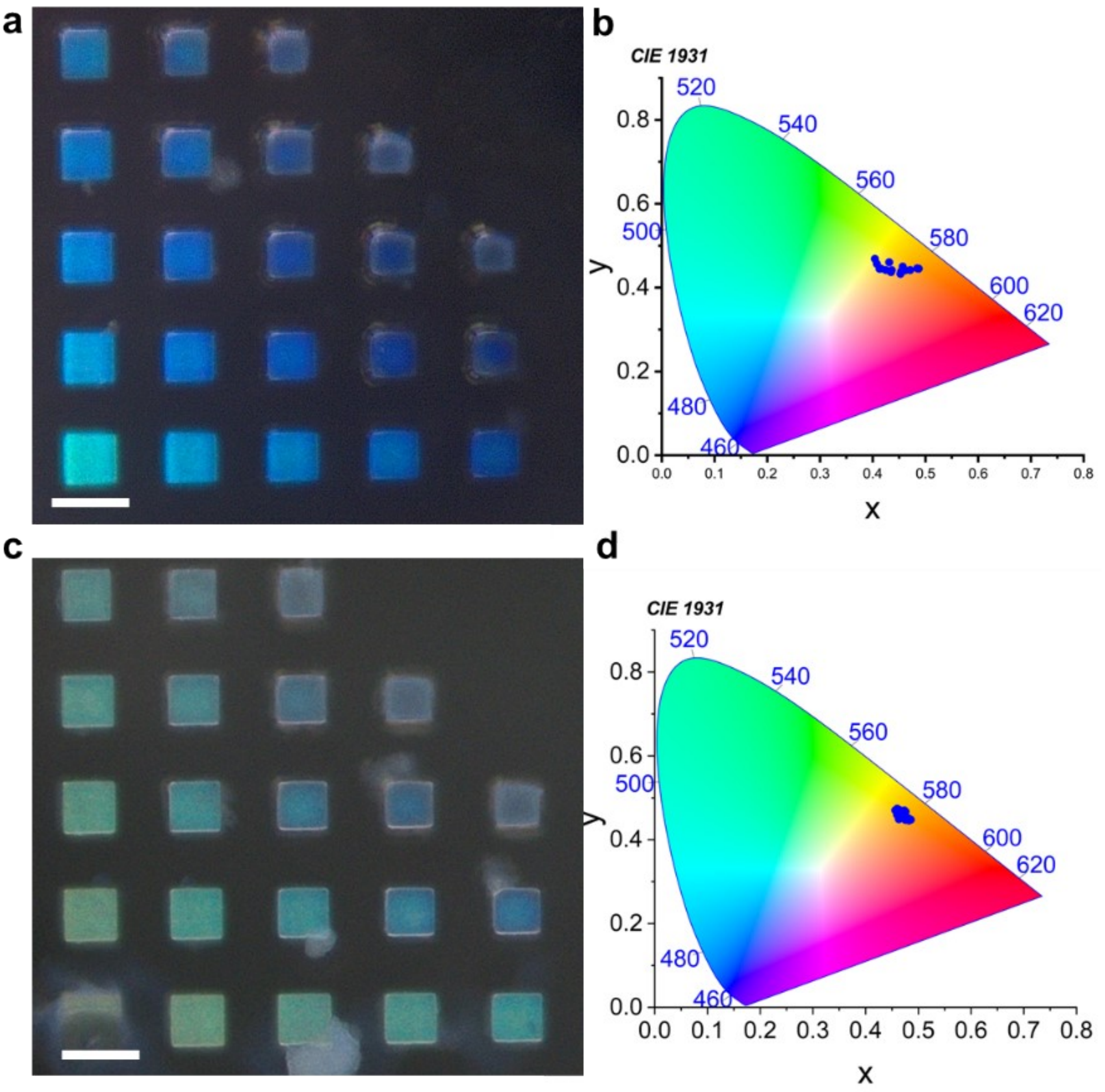

Figure S2. Structural colour printing using S1 and S2 nanoparticles composites. Both arrays were printed at SL = 0.6 μm and LP = 40%, with SS varied along the x-axis from 3000 to 15000 μm/s, and HD varied along the y-axis from 0.2 to 1.0 μm. a) Dark-field microscopy image of a 5 × 5 cube array printed using S1 nanoparticles and hydrated in deionized water (scale bar: 20 μm). b) CIE 1931 chromaticity diagram corresponding to the array in a. c) Dark-field microscopy image of a 5 × 5 cube array printed using PC2 and hydrated in deionized water (scale bar: 20 μm). d) CIE 1931 chromaticity diagram corresponding to the array in c.

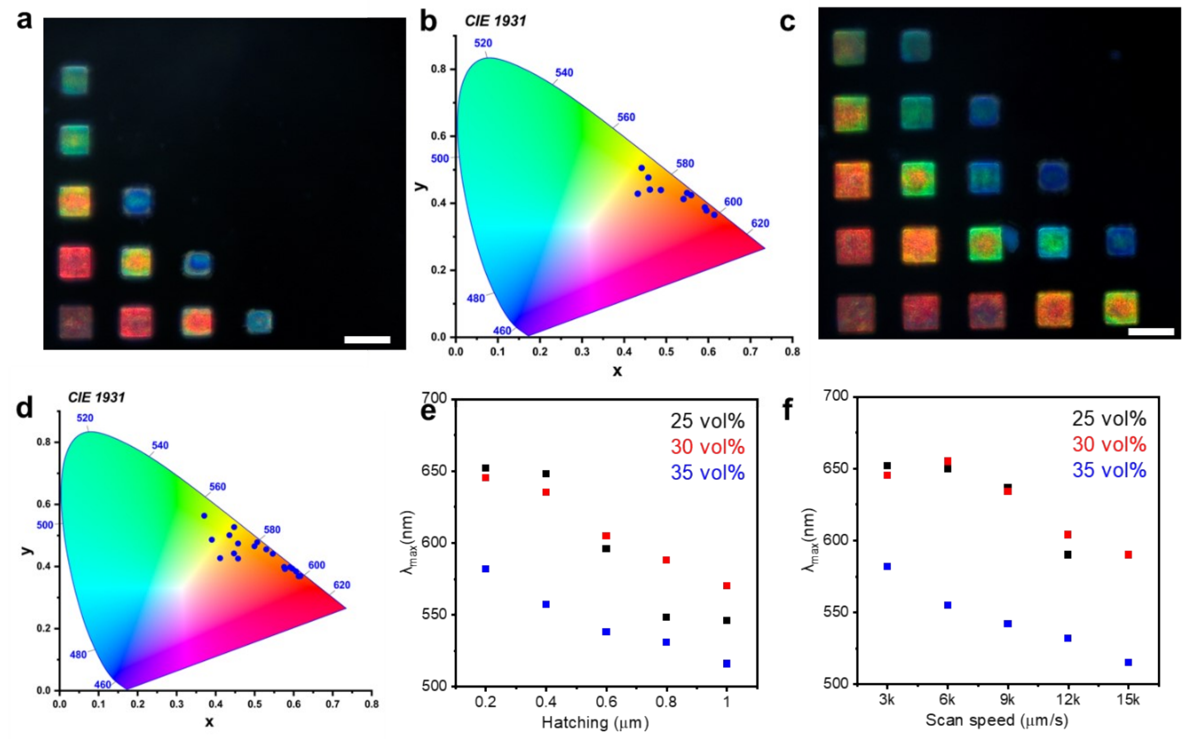

Figure S3. Influence of NP volume percentage on printed structures using PC3. a) Dark-field microscopy image of a cube array printed using 25 vol% NPs, under the same print conditions as Figure 3d (print parameters: SL = 0.6 μm, LP= 40%, x-axis represents SS from 3000 μm/s to 15000 um/s, while y axis represents HD from 0.2 to 1 um); b) CIE 1931 chromaticity diagram corresponding to panel a. c) Dark-field microscopy image of a cube array printed using 30 vol% NPs, under the same print conditions as Figure 3d (print parameters: SL = 0.6 μm, LP= 40%, x-axis represents SS from 3000 μm/s to 15000 um/s, while y axis represents HD from 0.2 to 1 um). d) CIE 1931 chromaticity diagram corresponding to panel c. e) Combined plot of λ_{max} vs hatching distance for 25%, 30%, and 35% vol at scan speed of 3000 μm/s. f) Combined plot of λ_{max} vs scanning speed for 25%, 30%, and 35% vol at hatching distance of 0.2 μm. Scale bar is 20 μm.

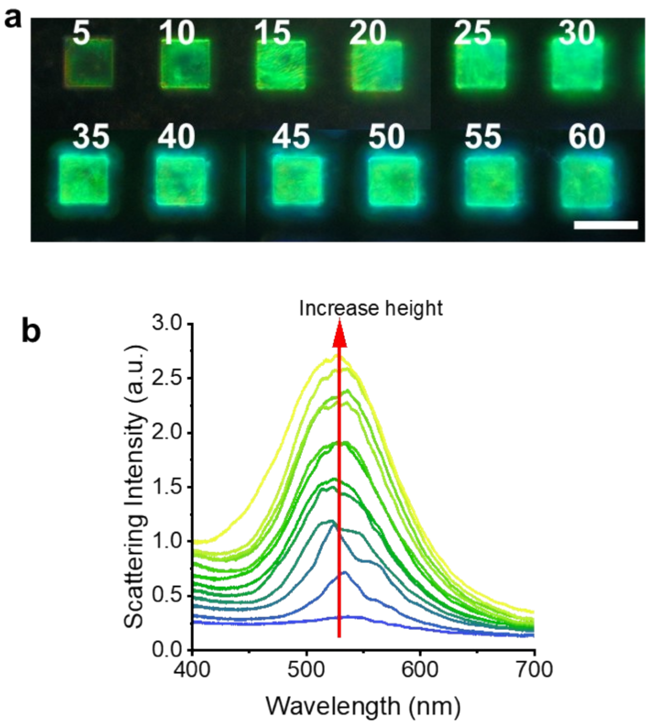

Figure S4. Influence of structural height on reflection band intensity. Cubes (20 μm × 20 μm × varying height) were printed with SL = 0.6 μm, HD = 0.6 μm, SS = 10000 μm/s, and LP = 70% in PC3. a) Dark-field microscopy image of the cube array with heights ranging from 5 to 60 μm in 5 μm increments. b) Corresponding reflectance spectra. Scale bar: 20 μm.

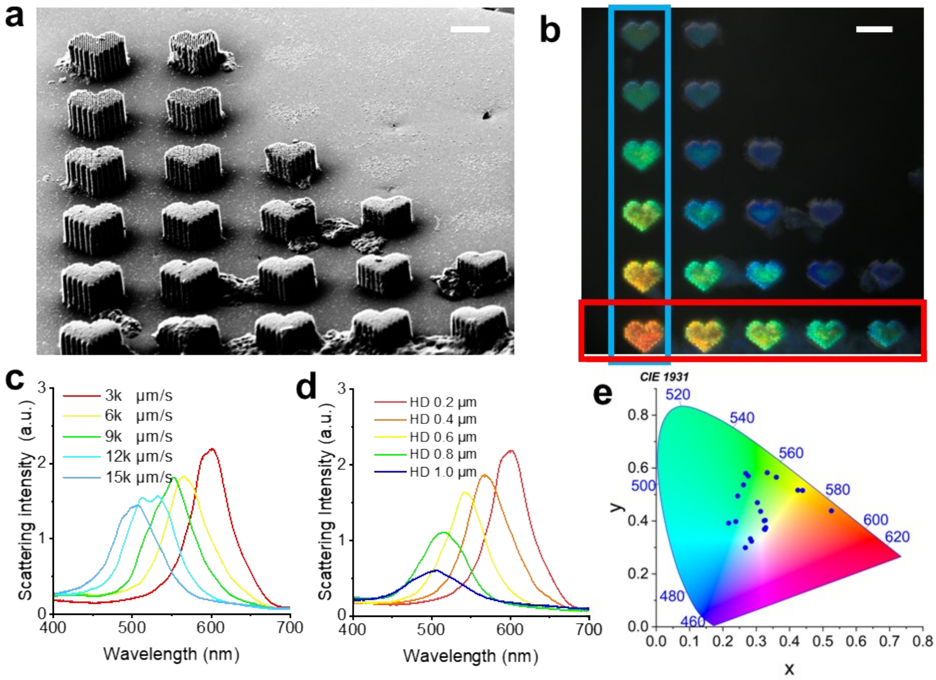

Figure S5. Design and fabrication of a wide colour-ranged microheart array fabricated in PC3. Each heart structure has dimensions of x = 32 μm, y = 24 μm, and z = 20 μm, printed at LP = 40% and SL = 0.6 μm. The x-axis represents SS from 3000 to 15000 μm/s, while the y-axis represents HD from 0.2 to 1.2 μm. a) SEM image of the microheart array. b) Corresponding dark-field microscopy image of array hydrated in DI water (scale bar: 20 μm). c) Scattering spectra of selected hearts highlighted in the red box, and d) in the blue box. e) CIE 1931 chromaticity diagram corresponding to the array shown in b.

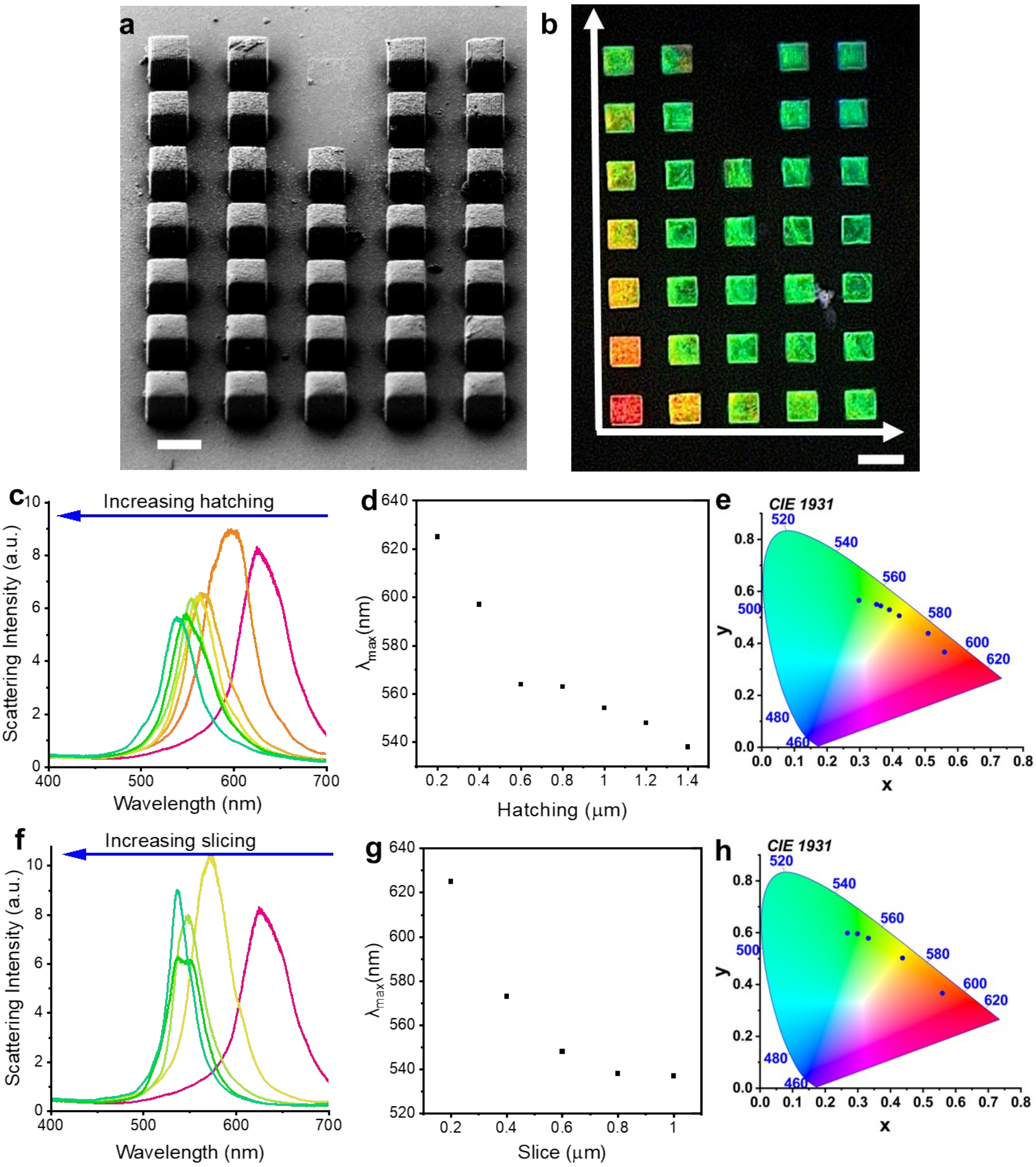

Figure S6. Influence of SL and HD on structural colour of microstructures fabricated in PC3 (print parameters: LP = 60%, SS = 10000 µm/s - the x-axis represents SL values ranging from 0.2 to 1.0 µm, while the y-axis represents HD values from 0.2 to 1.4 µm); a) SEM image, and b) dark-field microscopy image of the array hydrated in DI water. c) Scattering spectra of the column with fixed SL = 0.2 µm and increasing HD from 0.2 to 1.4 µm. d) Corresponding λ_{max} vs HD. e) CIE 1931 chromaticity diagram for panel c. f) Scattering spectra of the row with fixed HD = 0.2 µm and increasing SL from 0.2 to 1.0 µm. g) Corresponding λ_{max} versus SL. h) CIE 1931 chromaticity diagram for panel f. Scale bar is 20 µm.

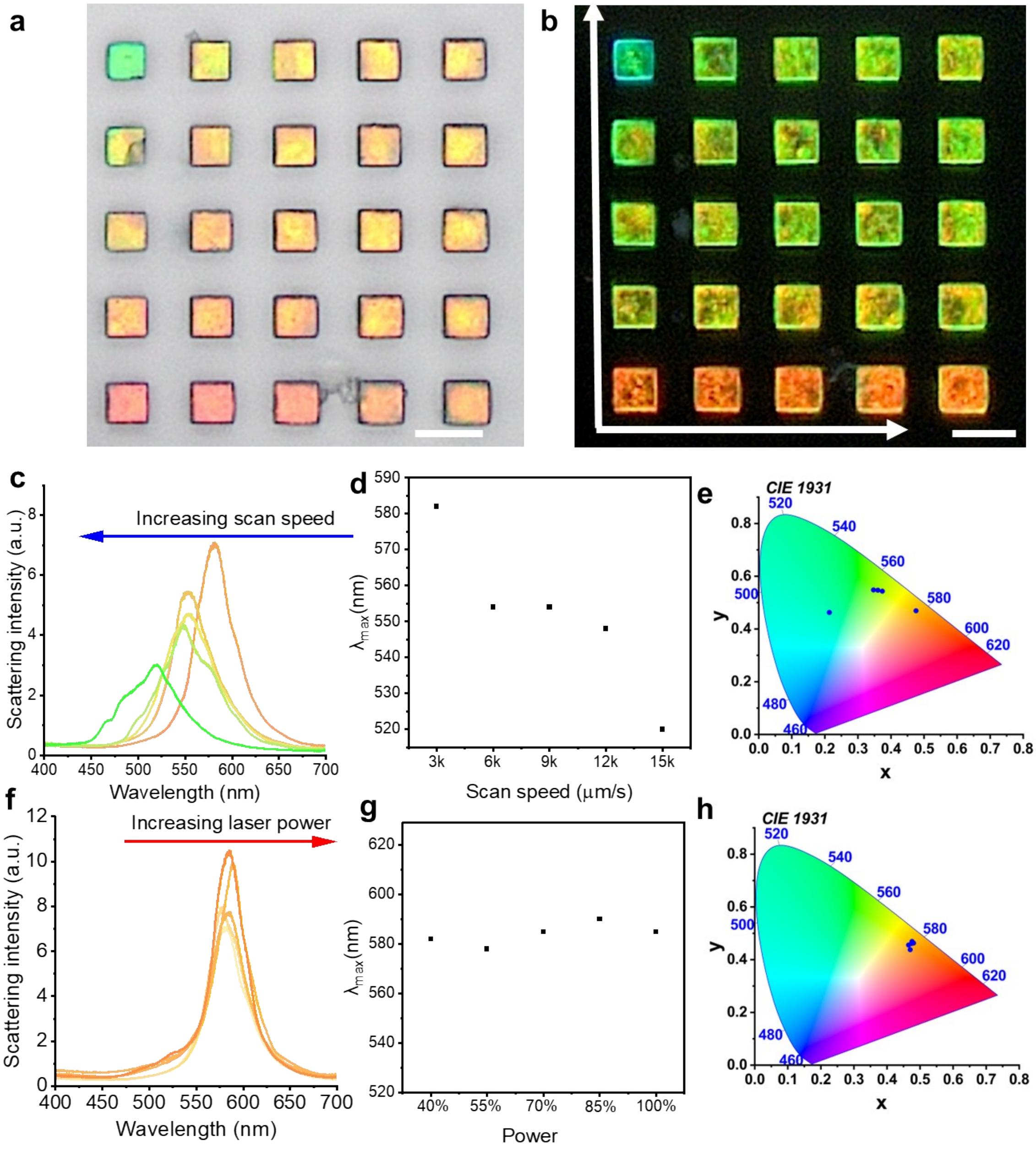

Figure S7. Influence of power and scan speed array on structural colour of microstructures fabricated in PC3 (print parameters: SL = 0.6 μm, HD = 0.2 μm, the x-axis represents LP from 40% to 100%, while the y-axis represents SS values from 3000 μm/s to 15000 μm/s); a) Bright-field microscopy image of the array in dried state. b) dark-field microscopy image of the array hydrated in DI water. c) Scattering spectra of the column with fixed LP = 40% and increasing SS from 3000 μm/s to 15000 μm/s. d) Corresponding λ_{max} vs SS. e) CIE 1931 chromaticity diagram for panel c. f) Scattering spectra of the row with fixed SS 3000 μm/s and increasing LP values from 40% to 100%. g) Corresponding λ_{max} vs LP. g) CIE 1931 chromaticity diagram for panel f. Scale bar is 20 μm.

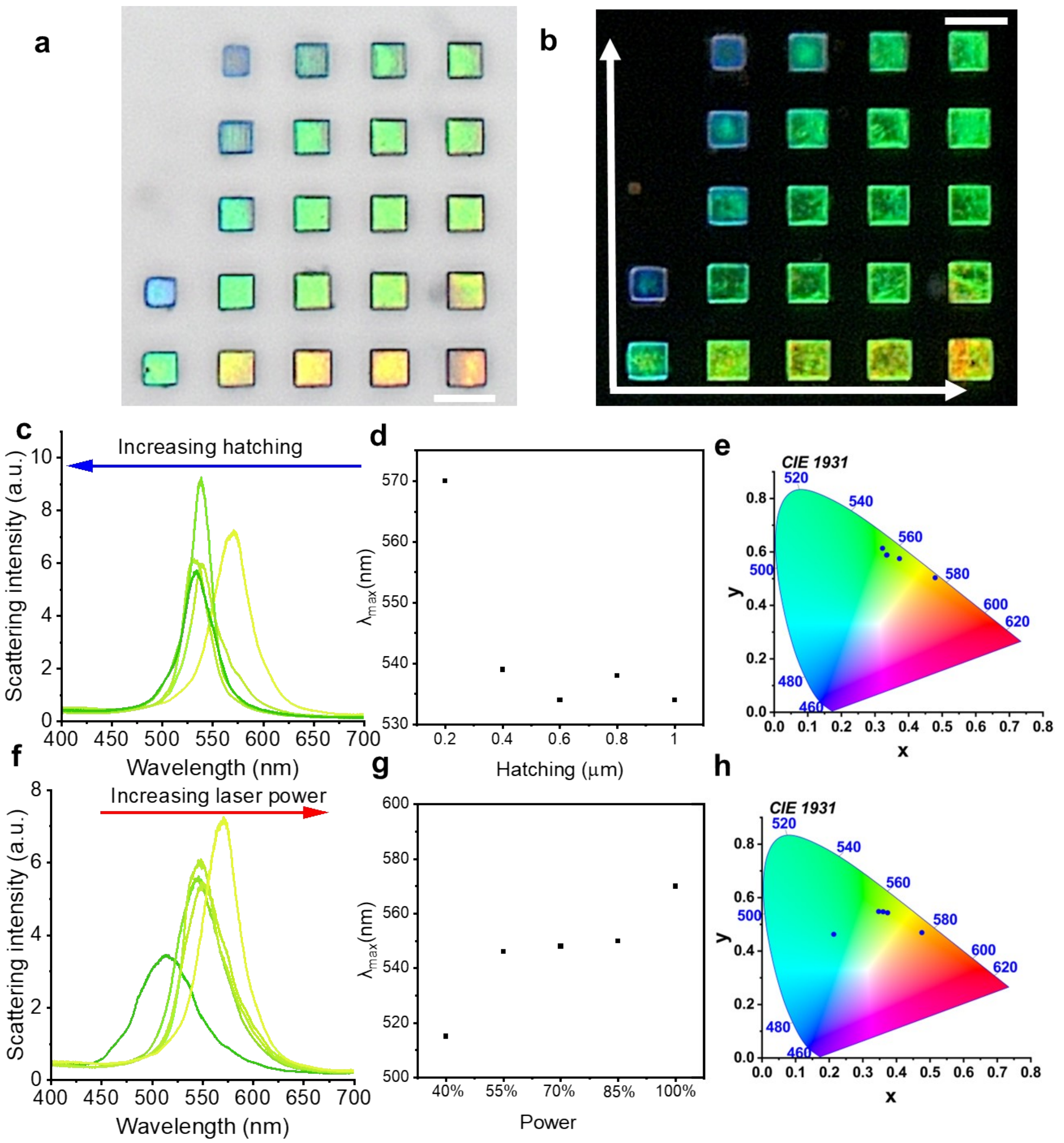

Figure S8. Influence of power and hatching array on structural colour of microstructures fabricated in PC3 (print parameters: SL=0.6 μm, SS = 15k μm/s, the x-axis represents LP values ranging from 40% to 100%, while the y-axis represents HD values from 0.2 to 1.0 μm); a) Bright-field microscopy image of the array in dried state. b) dark-field microscopy image of the array hydrated in DI water. c) Scattering spectra of the column with fixed LP = 40% and increasing HD values from 0.2 to 1.0 μm. d) Corresponding λ_{max} (nm) versus HD. e) CIE 1931 chromaticity diagram for panel c. f) Scattering spectra of the row with fixed HD at 0.2 μm, and increasing LP from 40% to 100%. g) Corresponding λ_{max} (nm) versus LP. h) CIE 1931 chromaticity diagram for panel f. Scale bar 20 μm.

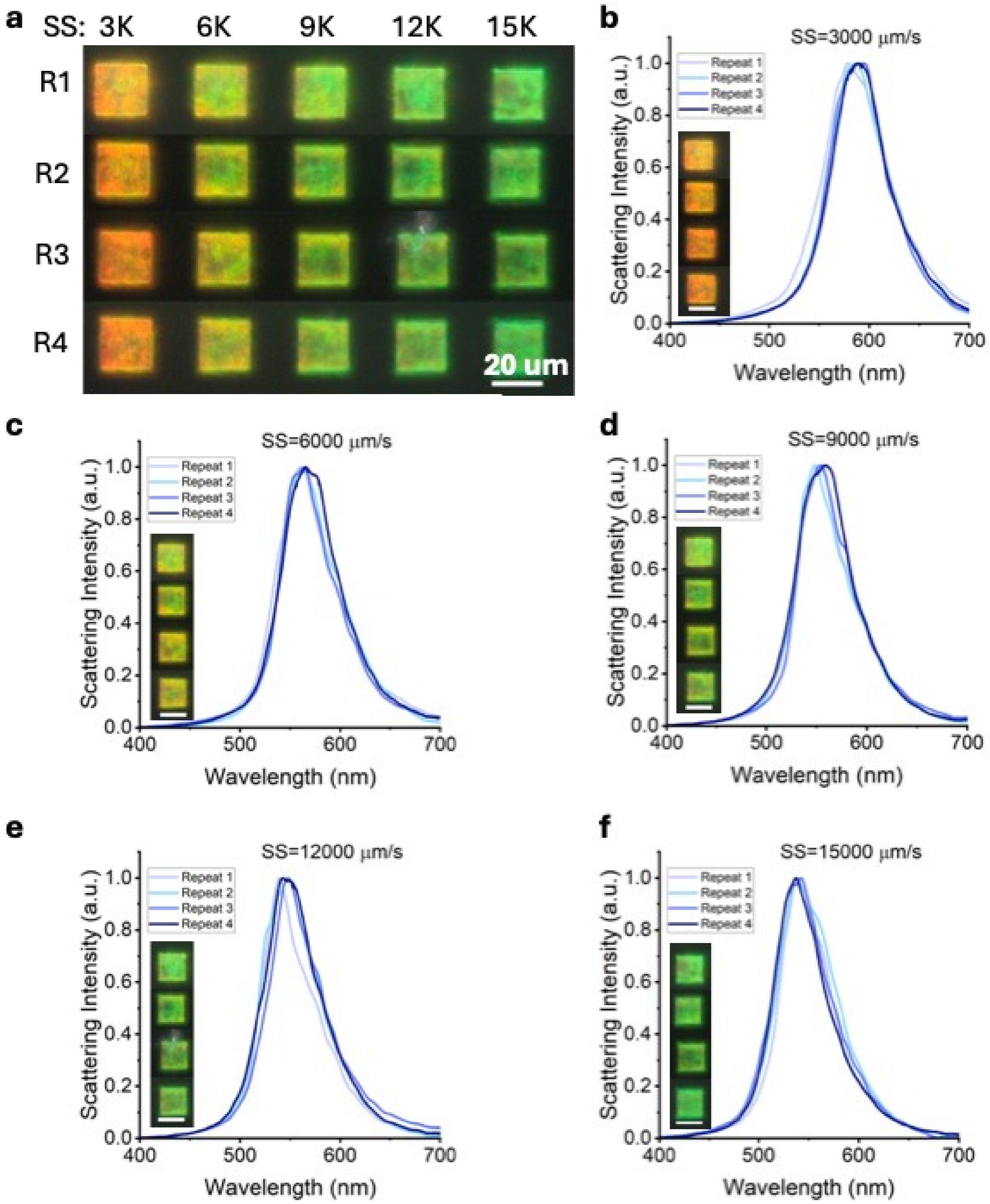

Figure S9. Reproducibility in the structural colour of microstructures fabricated in PC3. Array of micro-cubes of 20 μm by 20 μm fabricated at constant parameters SL = 0.4 μm, HD = 0.2 μm and LP = 40%, while varying SS across each row (R1 to R4) from 3,000 μm/s to 15,000 μm/s in 3,000 μm/s increments. a) Dark-field microscopy image of the array hydrated in DI water. Scattering spectra of four micro-cubes in the same column with SS = 3,000 μm/s (b); SS = 6,000 μm/s (c); SS = 9,000 μm/s (d); SS = 12,000 μm/s (e); SS = 15,000 μm/s (f). Scale bar 20 μm.

Table S2. Corresponding λ_{max} (nm) for each micro-cube shown in Figure S8a.

Scan Speed (SS, μm/s)	3K	6K	9K	12K	15K
R1(λ_{max})	579.3	560.4	554.4	539.2	537.7
R2(λ_{max})	585.5	565.1	548.2	548.2	537.7
R3(λ_{max})	594.7	565.1	553.2	548.2	538.2
R4(λ_{max})	589.1	565.8	559.4	543.3	542.0
Average (λ_{max})	587.1	564.1	553.8	544.8	538.9
St. Dev.	5.6	2.1	4.0	3.8	1.8

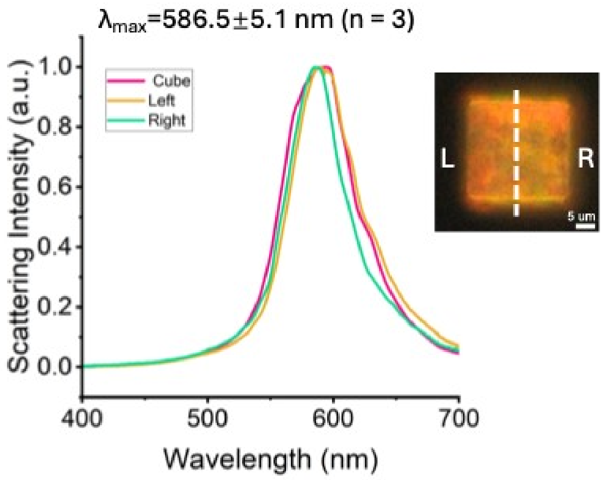

Figure S10. Scattering spectra the micro-cube shown in Figure S8, row R1, fabricated at LP = 40%, HD = 0.2 μm, SL = 0.6 μm, and SS = 3000 μm/s. Overlayed spectra measured for the entire cube, left half and right half of the cube, for comparison. Inset shows dark-field microscopy image of the micro-cube hydrated in DI water. Scale bar 5 μm.

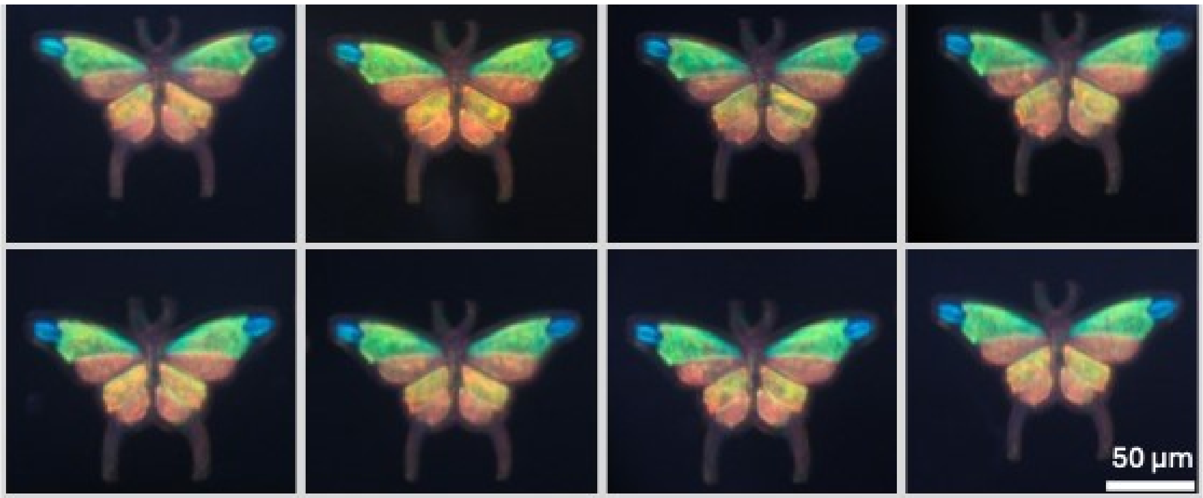

Figure S11. Reproducibility in the structural colour of microstructures fabricated in PC3. Dark-field microscopy images of butterfly structures hydrated in deionised water. The multi-coloured butterfly design shown in Figure 4a-d (fabricated using PC3 at 35 vol%), was reproduced across multiple substrates in multiple fabrication steps, to produce the eight butterfly structures shown, demonstrating the reproducibility of the colour printing. Scale bar 50 μm.

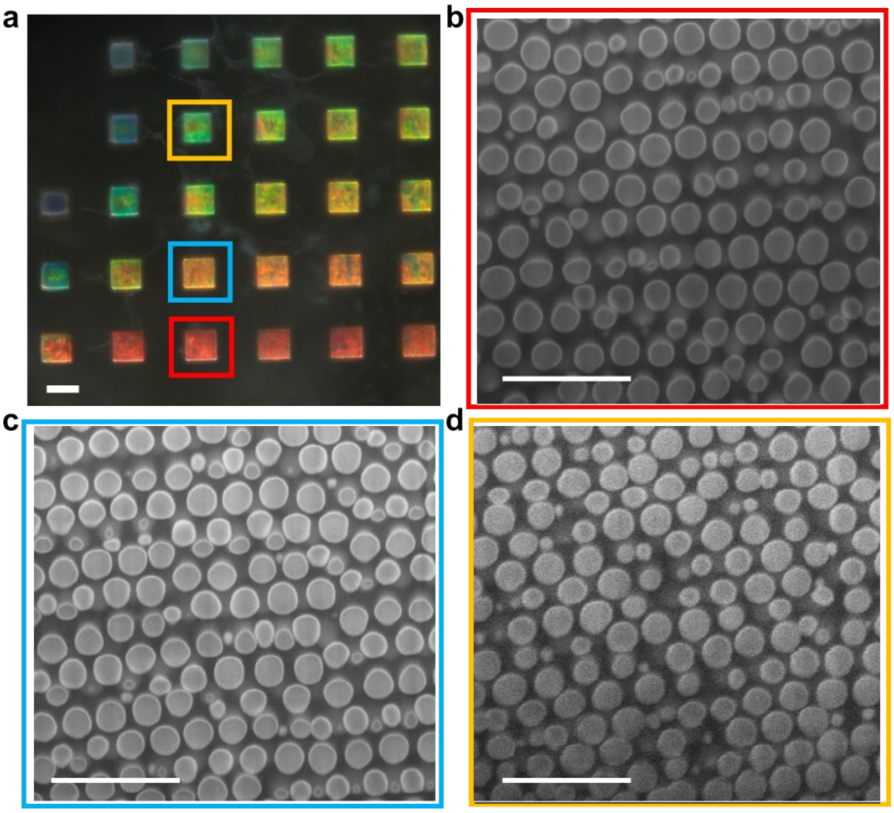

Figure S12. FIB-SEM analysis of the effect of hatching distance and laser power array. (Printing parameters: SL = 0.6 μm, SS = 1000 μm/s. The x-axis represents LP values from 40% to 90%, and the y-axis represents HD from 0.2 to 1.0 μm); a) Dark-field microscopy image of the array hydrated in DI water. b) FIB-SEM image of the structure highlighted in the red box (HD = 0.2 μm, LP = 60%). c) FIB-SEM image of the structure in the blue box (HD = 0.4 μm, LP = 60%). d) FIB-SEM image of the structure in the yellow box (HD = 0.8 μm, LP = 60%). Scale bars: 20 μm for the microscopy image and 1 μm for FIB-SEM images.

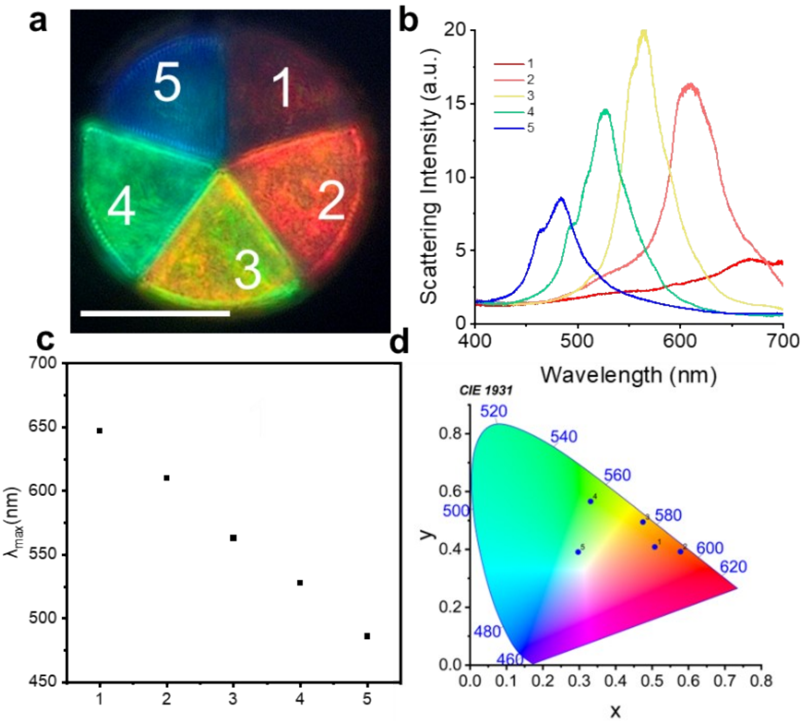

Figure S13. Fabrication of five-part colour wheel structure with distinct colours by tuning print parameters. (dimension: X = 26 µm, Y = 27 um, Z = 20 µm.) a) Dark-field microscopy image of colour wheel hydrated in DI water (scale bar: 20 µm). b) Corresponding scattering spectra. c) Corresponding λ_{max} (nm) for five regions. d) CIE 1931 chromaticity diagram for the colour wheel in a.

Table S3. DLW parameters used for the colours displayed in Fig S10.

Region	SL (µm)	HD(µm)	LP	SS (µm/s)
5	0.6	0.6	40%	9k
4	0.6	0.6	60%	10k
3	0.2	0.4	60%	10k
2	0.4	0.2	60%	10k
1	0.2	0.2	60%	10k

Simulation of dark-field scattering spectra

The reflected structural colour with normally incident light for well-ordered structures can be described by the Bragg-Snell law combined with the Maxwell-Garnett approximation for the effective refractive index as described by Augustine et al.[15], (*Small* 20.30 (2024): 2310058). The peak reflectance wavelength can be expressed:

$$\lambda_{max} = 2d_{111}n_{eff} = \left(\frac{\pi}{3\sqrt{2}\emptyset}\right)^{\frac{1}{3}} \times \frac{2\sqrt{6}d}{3} n_{eff}$$

where

$$\eta_{eff}^{2} = n_{polymer}^{2}\left[1 - \frac{3\phi(\eta_{polymer}^{2} - \eta_{silica}^{2})}{\eta_{polymer}^{2} + \eta_{silica}^{2} + \phi(\eta_{polymer}^{2} - \eta_{silica}^{2})}\right]$$

d_{111} is the distance between two neighboring FCC (111) planes and d is the particle diameter.

To explore the dependence of the dark-field scattering spectra on the nanoparticle diameter, volume fraction of nanoparticles and polymer refractive index, a rigorous numerical model is implemented using a commercial finite difference time domain simulation tool, Lumerical. The 3D NP structures are surrounded by absorbance monitors, a Mie source (TFSF source), and scattering monitors, as shown in the schematic below. 6 monitors were placed outside the light source to capture the signal. The total power signal from 5 monitors (except the one in transmission position) were divided by light intensity to normalize the scattering of cross section. The structure model was meshed with 5 nm resolution in 3 directions. Perfectly matched later boundary conditions were set in the x, y and z directions.

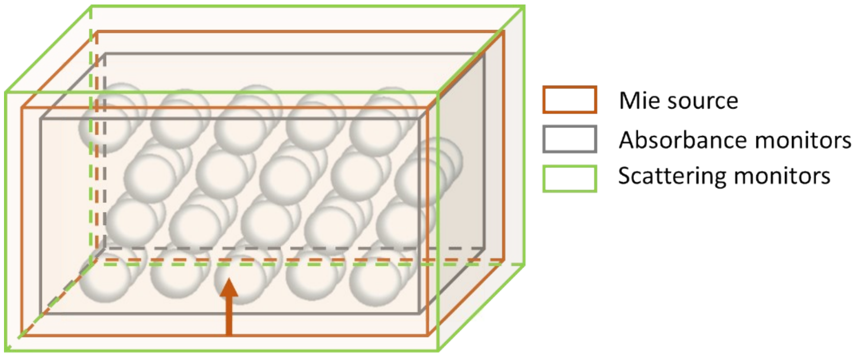

FDTD simulation model

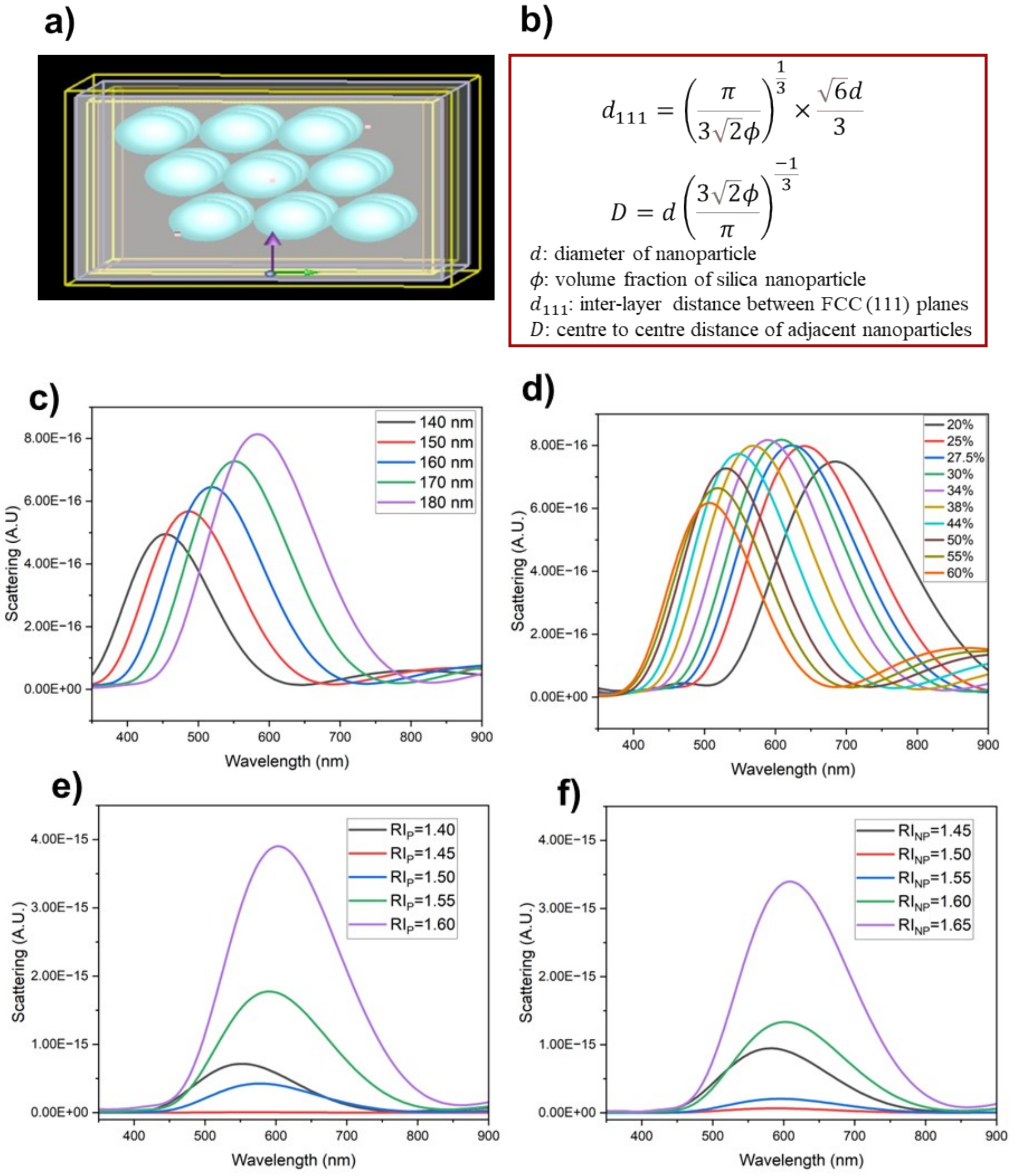

Figure S14. a) Schematic shows the FCC (111) structure used in Lumerical FDTD simulation, with (111) planes of the FCC structure stacking in z direction. b) Equations of interplane and inter-particle distances according to Bragg-Snell law. c) Simulated scattering spectra as the NP diameter was changed from 140 to 180 nm. d) Simulated scattering spectra as the volume fraction of nanoparticles increased from 20 to 60 %. e) Simulated scattering spectra as polymer matrix refractive index was changed from 1.4 to 1.6 holding RI_{NP} constant at 1.455. f) Simulated scattering spectra as NP refractive index was changed from 1.45 to 1.65 holding RI_P constant at 1.518.

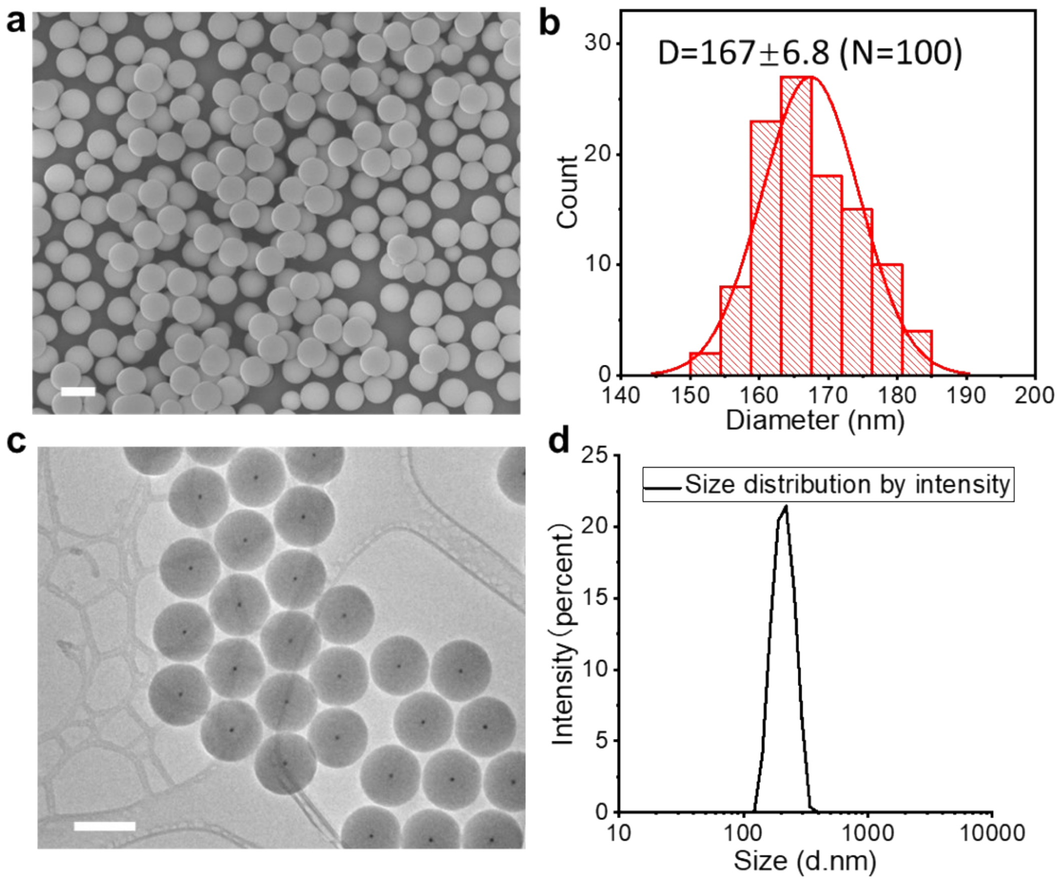

Figure S15. Characterisation of Au@Si NPs. a) SEM image. b) Size distribution measured from SEM images. c) TEM image. d) DLS measurement by intensity. Scale bar: 200 nm.

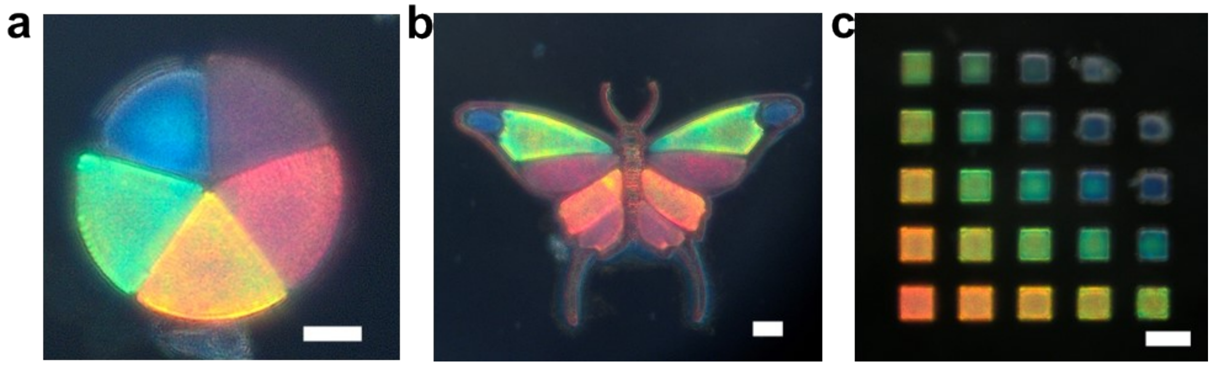

Figure S16. Dark-field images of 3D printed structures using Au@Si NPs at 35 vol%, hydrated in water. a) Five-part pie structure (printing parameters same as Figure S10d). b) Butterfly structure (printing parameters same as Figure 4). c) 5 × 5 microcube array (printing parameters same as Figure 3d). All scale bars: 20 μm.
